# A Comprehensive Study of Aluminum Anodization in Transition Modes

**DOI:** 10.3390/ma17143438

**Published:** 2024-07-11

**Authors:** Ilia Rozenblium, Yuliy Yuferov, Konstantin Borodianskiy

**Affiliations:** Department of Chemical Engineering, Ariel University, Ariel 40700, Israel; iliaro@ariel.ac.il

**Keywords:** anodization, AAO, high growth rate, nanoporous coatings

## Abstract

Anodization is a method to fabricate a tunable nanoporosity and thickness of alumina coating. This research is devoted to large-area hard anodization (HA), ultrahard anodization (UHA), and transitional modes. The phenomenon and challenges of UHA and the transition from HA are studied on large-area samples using linear-sweep voltammetry. The findings indicate that a uniform large-area thick coating can be achieved by utilizing pre-UHA modes. The study’s results indicate that UHA leads only to coatings with non-uniform thickness in large-area anodization. The peculiarities of pre-UHA are studied using different temperatures (0, 5, 10, and 15 °C) and processing times (1, 2, 4, 6, and 12 h) in a 0.3 M oxalic acid electrolyte. The current study shows the possibility for the fast growth of thick nanoporous alumina up to 235 ± 4 µm for only 12 h.

## 1. Introduction

Anodization is a common surface treatment process that is a robust, cost-effective approach to providing a surface with unique properties, such as enhanced corrosion resistance, improved wear resistance, and decorative coatings. Anodized surfaces also improve coating adhesion to paint, and may serve as dielectric materials. The anodization product, anodized aluminum oxide (AAO), has raised substantial scientific and technological interest due to its diverse applications as templates [1,2,3], membranes for filtering and separation [4,5], drug delivery [6], functional layers for composites [7], sensors, and biosensors [8,9,10], etc.

Over several decades, scientists have extensively studied the phenomenon of AAO formation to elucidate the mechanism of self-organized growth of the porous layer. The electrochemical process of anodization releases electrons from the aluminum anode, creating aluminum ions on the surface. The aluminum ions then react with the oxygen carriers to form the oxide layer on the metal’s surface. At the cathode, the hydrogen ions undergo reduction by receiving electrons, forming hydrogen gas. The electrolyte solution releases oxygen carrier ions, which tend to combine with aluminum ions, resulting in the development of AAO on the metal’s surface. Sulka described a more detailed mechanism [11] where AAO growth models were well described. Later, the origin of self-organization in AAO films was characterized by Rayleigh–Benard convection cells in combination with an imaginary flow of colloidal particles that form the walls and pores of AAO [12].

The overall equation of the process is given below:

2Al + 3H_2_O → Al_2_O_3_ + 3H_2_ (1)

Self-ordered AAO membranes with specific structure parameters of close-packed hexagonal cells can be obtained under conventional two-step mild anodization (MA) in different acidic electrolytes such as H_2_SO_4_, H_2_C_2_O_4_, and H_3_PO_4_. It was shown that the shape and arrangement of the AAO membranes are controllable and affected by the electrolyte’s composition [13], temperature [14], and applied process voltage or current [14]. Alternatively, AAO membranes were obtained under high anodizing voltage (hard anodization (HA)), a process in which the oxide growth rate is significantly higher than conventional MA. However, ref. [15] shows that anodization in the transfer region from MA to HA (60–80 V) is not perfectly stable and presents inconsistencies in the AAO growth rate and pore arrangement. The authors reported that at a lower temperature (7 °C), a thick AAO film was produced, although it was of poor quality and had disordered pore formation. Furthermore, they also revealed that the longer process (with the same anodization conditions) decreased the AAO film thickness. This transfer region from MA to HA corresponds to the mixed regime reported in the paper [16]. Here, the authors illustrated three growth modes: diffusion, mixed, and kinetic limitations, equivalent to mild, mixed, and hard anodization modes, respectively. It was shown that the growth of alumina with a sample area of 8.0 cm^2^ can reach 120–150 V in a 0.3 M oxalic acid electrolyte, depending on the electrolyte temperature. In addition, the maximal voltage decreases with an increase in temperature. Earlier, Lee et al. showed the pre-anodization application to form a thin oxide layer that suppresses breakdown effects and enables the growth of the uniform oxide film at high voltages with surface patterning by the nano-imprinted mold [17]. Recently, ultra-hard anodization (UHA) modes were discovered, partially enclosed in [18]. However, the application of UHA is strongly limited by complex equipment, and only a small treated area of 0.5 cm^2^ was successfully uniformly anodized, mostly due to the strict hydrodynamic conditions that still require extensive research on large areas. Some of these large-area effects will be disclosed in the current study by anodization in the transitional region. Moreover, the heat generated during UHA requires sufficient specific cooling and hydrodynamic conditions.

This study aims to form a uniform nanoporous alumina membrane with a high and uniform thickness (>200 µm) on a large-area sample utilizing pre-UHA modes, which may be easily scaled up to be utilized on large-area substrates. Additionally, this study focuses on the influence of process parameters such as electrolyte temperature, anodization time, and different voltages and peculiarities of the pre-UHA modes on the thickness and nano-structured geometry of the AAO.

## 2. Experimental Details

### 2.1. Anodization Pre-Treatment

Highly pure aluminum (99.99%, Testbourne Ltd., Basingstoke, UK) with a rectangular shape and size of 20 × 40 × 1 mm was used as a template. The initial Al surface was pre-treated using wet grinding and polishing by #600, #1200, and #2500 SiC papers using a LaboForce-100 (Struers Ltd., Ballerup, Denmark) machine. Then, substrates were washed with de-ionized water, degreased with acetone and ethanol, and dried with air.

### 2.2. Anodization

**Figure 1:**
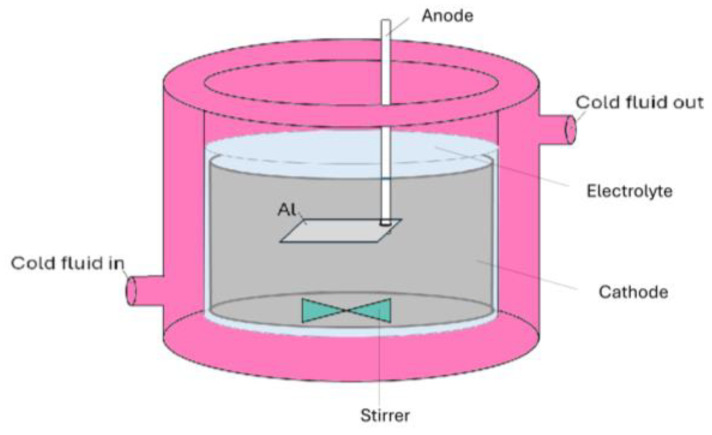
Schematics of the electrochemical cell used for the Al anodization.

**Figure 2:**
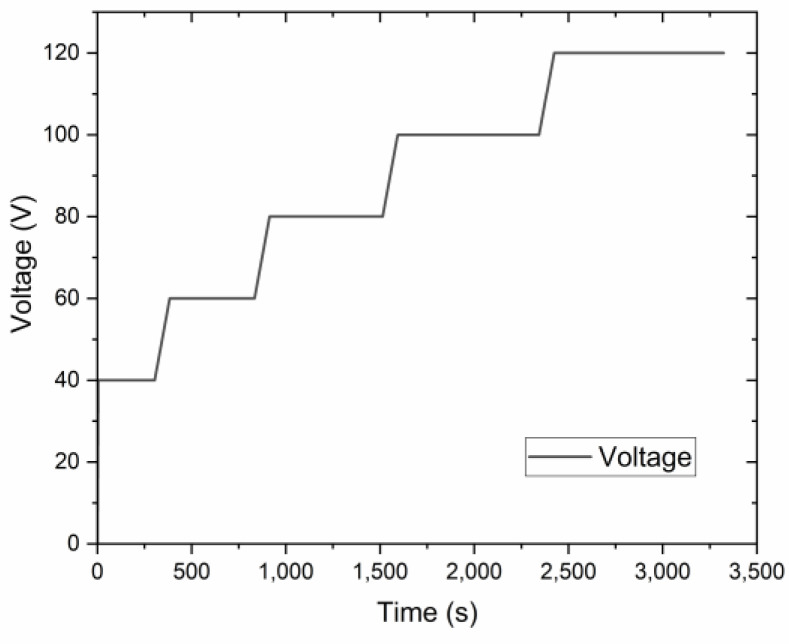
The voltage-time curve of the pre-anodization procedure.

Al templates with an area of 17.2 cm^2^ were anodized in a 0.3 M oxalic acid (purity ≥ 99.5%, Fisher Chemical, Loughborough, UK) electrolyte using a programmable DC power supply, IT6006C-500-40 (Itech Electronic Co. Ltd., New Taipei City, Taiwan). The anodization process was carried out utilizing a 1 L double-jacketed glass reactor (d = 9.5 cm, h = 14 cm) filled with 0.5 L oxalic acid electrolyte equipped with a cooling system WBL-700 (MRC, Swindon, UK), shown in Figure 1. The cathode material was stainless steel with an area of approx. 184 cm^2^ (d = 9 cm, h = 6.5 cm). The distance between the magnetic stirred bar and the anode was approx. 3.5 cm. Anodization was conducted at a range of voltages up to 140 V. The pre-anodization was carried out according to the procedure shown in Figure 2, with a voltage sweep rate of ~0.25 V/s up to the final anodization voltage. The experiments were conducted at various temperatures (0, 5, 10, and 15 °C), with a time variation from 1 h to 12 h and a stirring rate of 400 RPM.

### 2.3. Characterization

The effect of different pre-anodization layers was studied using linear sweep voltammetry (LSV), which allows for the evaluation of its effect on the anodization mode. Different pre-anodized layers were grown, as shown in Table 1. After the pre-anodization, we used the anodic polarization test using LSV with a sweep rate of 0.05 V/s (Figure 2 and Table 1).

The thickness of the AAO membranes was measured using an optical digital microscope system RH-2000 (Hirox Inc., Tokyo, Japan). The nanostructure morphology and the thicknesses of the samples were characterized using scanning electron microscopy (SEM) JSM 6510 (Jeol Inc., Tokyo, Japan) and MAIA 3 (Tescan Inc., Brno, Czech Republic). SEM images of the surface and cross-section of coatings were analyzed with freeware ImageJ2 software, version 1.54g.

## 3. Results and Discussion

**Figure 3:**
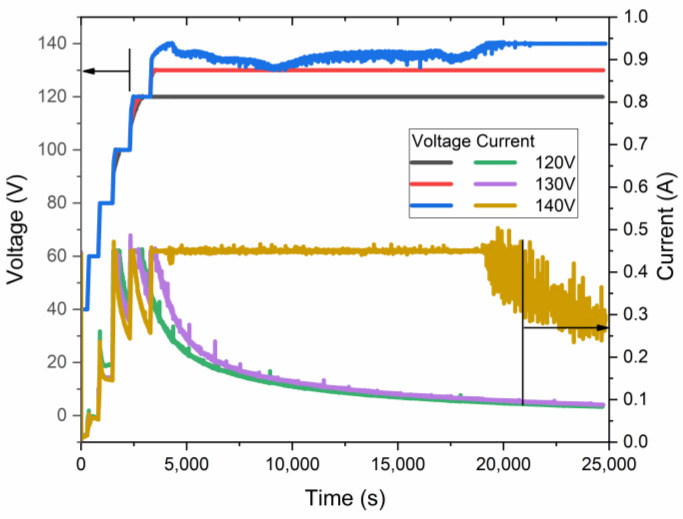
Voltage–current–time relationship of samples anodized at 0 °C at different maximal anodization voltages.

The samples of AAO were obtained using anodization in a wide-spread electrolyte of an aqueous solution of 0.3 M H_2_C_2_O_4_. The process was conducted in HA and pre-UHA modes using a pre-anodization procedure. This procedure ensures the growth of a thin nanoporous layer, which consequently protects the sample from an anodic burning effect and breakdown [17]. The current-time and voltage-time of the anodization process in near UHA modes at 0 °C are shown in Figure 3.

As revealed in voltage–current–time curves, the highest voltage, quite below 140 V, was achieved for the samples obtained at nearly 0 °C. This sample was anodized for less than 5 h in galvanostatic mode, limited by 0.45 A and a voltage of ~135 V. Such a current limit is necessary because of the significant heating of the sample during the initial growth of AAO. Only after 5 h of anodization does the voltage reach an almost constant value of 140 V. The authors of [17] have shown slightly higher anodization voltages, up to 150 V, under similar conditions, which may be addressed to different cell geometry, cooling procedures, pre-anodizing procedures, surface pre-pattering, and hydrodynamic conditions. However, in the current work, the sample was placed coaxially in a large-volume double-jacketed reactor with a magnetic stirrer below it. This setup may further facilitate the treatment of a larger sample area.

**Figure 4:**
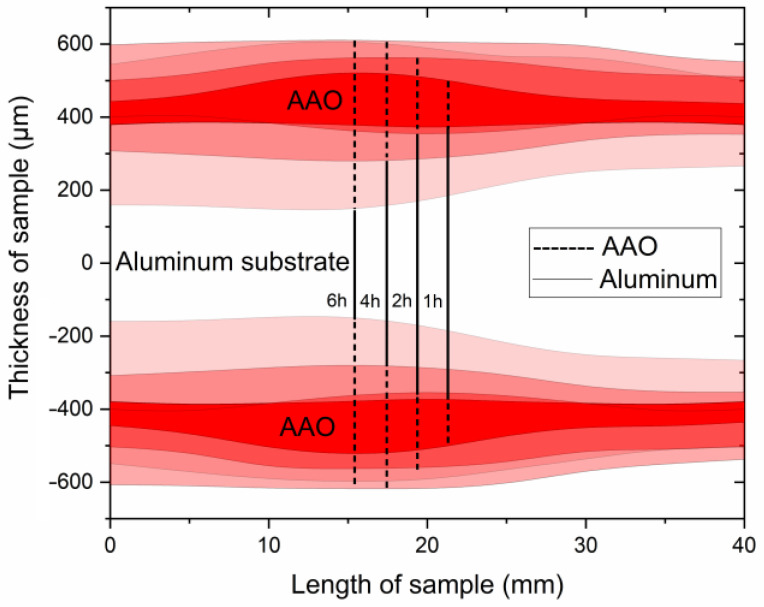
Schematics of the AAO thickness distribution obtained at 0 °C and 140 V at different anodization times.

**Figure 5:**
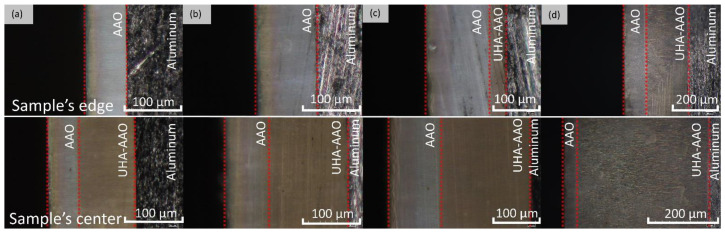
Optical cross-section micrographs of the AAO thickness distribution obtained at 0 °C and 140 V at different anodization times: (**a**) 1 h, (**b**) 2 h, (**c**) 4 h, and (**d**) 6 h.

As shown in Figure 4, samples obtained at 140 V exhibit a strong non-uniformity in coating thickness across the sample. This non-uniformity may be attributed to the non-uniform cooling and different hydrodynamic conditions along the sample surface during anodization at a high voltage that might be described as a transitional borderline to UHA mode. Observation of cross-section micrographs in Figure 5 revealed that these coatings consist of several layers. The first layer is addressed to the pre-anodization, creating a gradient color on the surface of the sample (left side on the cross-section). The next gray-colored layer is addressed to the common AAO coating. Then, the brown one probably belongs to the UHA-AAO layer, which was grown under the AAO layer during the galvanostatic mode.

As can be seen from Figure 4 and Figure 5, the thicknesses of AAO and UHA-AAO layers increased with the duration of the anodization process in the galvanostatic mode. The UHA-AAO layer was grown more intensively than the common AAO. Moreover, this layer also spreads with an increase in anodization time from the central zone to the edge of the sample.

**Figure 6:**
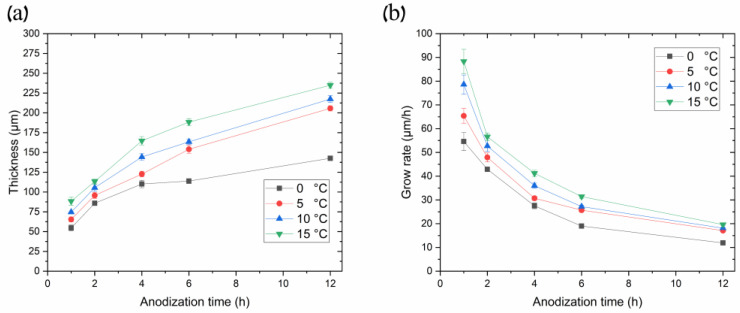
(**a**) Curves of thicknesses and (**b**) growth rates of AAO porous films synthesized in 0.3 M oxalic acid at 120 V, utilizing different temperatures as a function of anodization time.

We believe the thicker part of the coating most probably grew in conditions close to UHA, as agreed with [18]. However, the authors of [18] reported uniform AAO coating obtained on a small anodizing area of less than 1 cm^2^. This highlights the primary challenge of achieving the UHA mode on large-area samples. This behavior may be attributed to different heating exchanges, where the center of the sample is heated more than the edges, as clearly illustrated in the plot showing the distribution of AAO thickness (Figure 4). The thicker oxide layer mostly leads to a reduction in the heat exchange of AAO. This non-uniform thickness follows temperature irregularities in the reaction zone, resulting in a non-uniform growth rate distribution along the sample. Thus, rapid fabrication of a large-area, thick, and uniform AAO coating requires lower voltages or a so-called pre-UHA mode. This approach yields a growth rate of 19.6 ± 0.3 µm/h (120 V, 12 h, 15 °C) in long-term anodization, as shown in Figure 6., compared to common MA rates of 12.9 µm/h (45 V, 8 h, 20 °C) [19] or HA rates of 12.4 µm/h (80 V, 10 h, 10 °C) [15].

Figure 6 illustrates the relationship between AAO thickness and its growth rate over anodization time at different processing temperatures in the pre-UHA mode (anodization voltage 120 V). Figure 6a demonstrates a sharp increase in the AAO thickness during the initial processing hours, followed by a gradual slope smoothing, as also observed in [17,18]. This behavior differs from processes conducted at lower voltages, such as in MA, where the slope of the thickness versus anodization time remains almost linear during long-term processing, up to 16 h [19].

**Figure 7:**
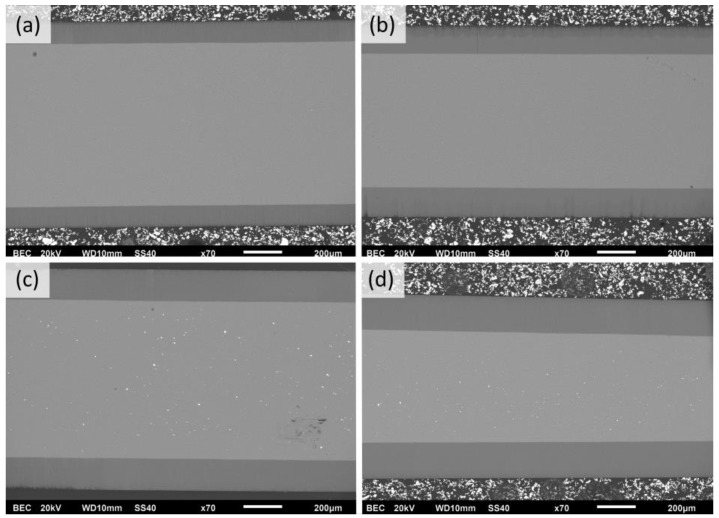
SEM cross-section micrographs of AAO obtained in potentiostatic mode of 6 h of anodization at 120 V in electrolytes with the following temperatures: (**a**) 0 °C, (**b**) 5 °C, (**c**) 10 °C, and (**d**) 15 °C (scale marker is 200 µm).

The evaluation of Figure 6b revealed that the higher the process temperature, the higher the growth rate over all ranges of anodization times. That depicts the common growth-rate–temperature relationship in common MA and HA, which is supported by [14,16,20,21]. The cross-section SEM images of AAO coatings obtained during 6 h of anodization at 120 V in different temperatures are presented in Figure 7. The observations revealed that the thickness of AAO coating increased from 113.8 ± 3.2 to 188.3 ± 4.4 µm with the temperature rise from 0 to 15 °C.

**Figure 8:**
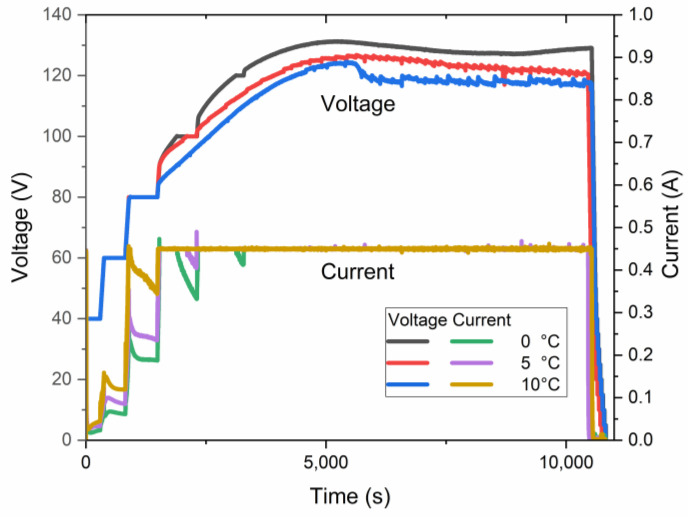
Voltage–current–time relationship of samples anodized at a target voltage of 140 V.

Simultaneously, an increase in the electrolyte temperature facilitates a decrease in the maximum anodization voltage, as shown in Figure 8. The curves in this plot present the electric behavior of three samples anodized at a target voltage of 140 V at different temperatures. Although the processes did not reach the target voltage, the electrolyte temperature affects the maximum voltage for anodization. The maximum constant pre-UHA voltage of ~128 V, ~127 V, and ~125 V was reached at 0 °C, 5 °C, and 10 °C, respectively.

**Figure 9:**
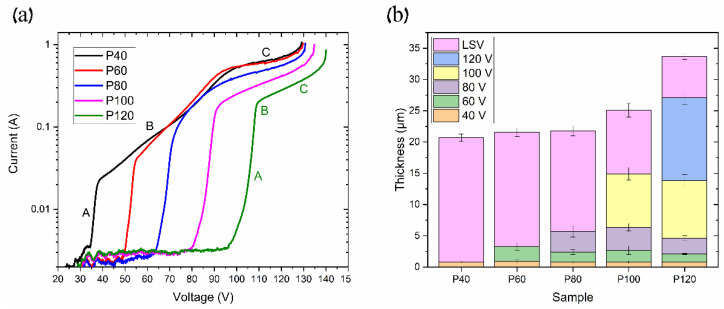
Samples with different pre-anodization procedures: (**a**) linear sweep voltammetry (LSV) curves and (**b**) thicknesses of AAO layers at each pre-anodization procedure.

It should be noted that the maximum voltage is also associated with the pre-anodization procedure, which may be evaluated using an anodic polarization or linear sweep voltammogram (LSV). The obtained LSV of the pre-anodization at 0 °C is shown in Figure 9a.

The LSV test was carried out on samples with different pre-anodization procedures, as described in Table 1. The obtained curves show the following three typical modes of anodization: kinetic, mixed, and diffusion [16,22,23]. The kinetic and diffusion modes may be fitted by line in Figure 9a. In area A (MA), the anodization rate is limited by the chemical and electrochemical processes’ kinetics at the pore bottoms under the applied electric field. Area B (mixed mode anodization) includes the diffusion limit of ions through the AAO pores, along with the kinetics of ion transport at the solid/electrolyte interface. In contrast, area C (HA) describes the ion diffusion limit due to the high ion content at the AAO pore bottom, which is also agreed upon in [16]. The continuation of area C, where the linear region ends, represents the maximum voltage before the anodization process transitions to UHA or even the plasma electrolytic oxidation (PEO) zone. As seen in Figure 9a, the pre-anodization procedure can shift the anodization modes to higher voltages and increase the maximal voltage. Thus, in the P40, P60, P80, P100, and P120, the kinetic mode begins at 35 V, 50 V, 65 V, 80 V, and 95 V, respectively. Furthermore, we observe that the kinetic limit area (area A) increases with the increasing thickness of the coating developed during the pre-anodization procedure. Concurrently, the mixed mode range decreases with the longer pre-anodization procedure, as indicated by the change in area B on the curves from P40 to P120. It can be seen in Figure 9b that the layer thickness at each pre-anodization step is almost similar across all of the samples. However, the thickness of the LSV coating layer reduces as more pre-anodization steps are applied. This phenomenon may be addressed to the reduction in the current density with the anodization voltage, as seen in Figure 9. Consequently, pre-anodization introduces additional growth limitations, shifting the diffusion mode to higher values. This permits using a higher anodization voltage, the so-called pre-UHA mode, especially at elevated temperatures where the voltage range for the pre-UHA is broader, resulting in a higher growth rate than HA at lower voltages [15].

Such a study of LSV allows for the disclosure of the UHA phenomena by considering the sample anodized at 0 °C, which has not reached a target voltage of 140 V, as seen in Figure 3. This behavior is addressed to the transfer to the galvanostatic mode, previously explained as a local heat generation, resulting in a high voltage employed with a local increase in electrolyte temperature at the bottom of pores in the reaction zone. This behavior directly affects the mode transition with a shift of modes at rising temperatures. An induced local UHA competes and is parallel to HA or pre-UHA outside the local UHA zone. Although the process finally transfers to the potentiostatic mode, the thickness of AAO remains non-uniform, as illustrated in Figure 4. However, samples anodized at 120 V and 130 V reached the target voltage. They showed a potentiostatic behavior with uniform oxide growth on the aluminum surface in a wide range of temperatures from 0 to 15 °C.

**Figure 10:**
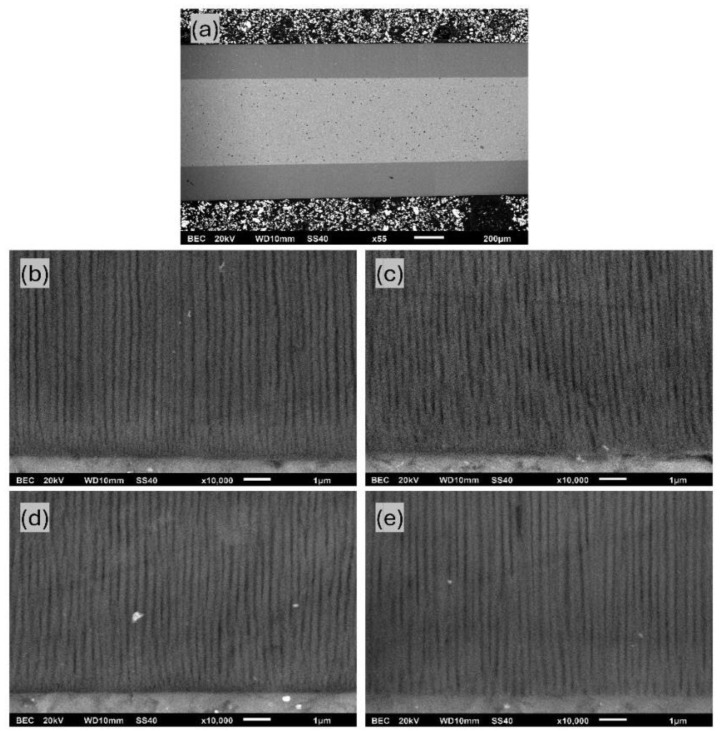
SEM cross-section micrographs of (**a**) AAO layer obtained at 15 °C and 12 h, and AAO samples anodized at 10 °C utilizing the following various anodization times: (**b**) 1 h, (**c**) 2 h, (**d**) 4 h, and (**e**) 12 h.

The dependence of the AAO thickness and growth rate upon temperature and anodization time in the pre-UHA mode has been studied. Figure 6a shows that most AAO coatings were obtained between 0 and 15 °C, utilizing an anodization of 1 to 12 h. The thinnest achieved AAO coating was 54.6 ± 3.8 µm (0 °C, 1 h), and the thickest was 234.9 ± 3.7 µm (15 °C, 12 h), as evaluated in a SEM cross-section micrograph, as shown in Figure 10a. Implementing the pre-UHA enables the achievement of a uniform oxide layer and a high AAO growth rate of ~90 µm/h on a large-area sample (17.2 cm^2^) during short-term anodizing, as shown in Figure 6b. The growth rates and thicknesses in short-term anodization were also much higher compared to those the presented in [16]. This finding may be attributed to the lower thickness of the pre-anodized layer at 40 V (~1 μm) in comparison to the thickness of ~5 μm 40 V reported in [16]. The thickness of AAO coatings obtained at 120 V and 0 °C was also lower (up to 40 µm) over the same anodization time range, compared to 4 h of anodization at 140 V and 1 °C in reference [17]. However, unlike in [17], the current study does not require the use of a special cooling planar cell, which significantly restricts the anodizing area. In summary, a target anodization voltage of 120 V was selected for the pre-UHA, as this value demonstrated stable potentiostatic anodization across all electrolyte temperatures (0–15 °C). Increasing the process voltage to 130 V posed challenges in maintaining the potentiostatic process at higher temperatures of 10 and 15 °C, leading to a transition to local UHA, as previously observed. It should be noted that pre-UHA requires constant cooling, as any disruptions in cooling may cause a transition to local UHA.

The average pore diameter for different anodization processes is listed in Table 2. Calculations were performed using SEM cross-section micrograph images, such as Figure 10b–e, which is related to coatings at 12 h. As evident from the listed values, higher temperatures and longer anodization times lead to an increase in the pore diameter. This behavior is controllable and consistent with those previously reported in [14,24]. The largest pore diameter of 155.9 ± 17.0 nm was obtained using anodization at 15 °C for 12 h.

## 4. Conclusions

In this study, large-area AAO were produced utilizing a comprehensive range of experimental conditions.

A deeper understanding of UHA phenomena was achieved through LSV analysis and anodization in UHA conditions, indicating that this process is very similar to HA. Here, under critical conditions, the growth rate of nanoporous oxide was increased due to the high local temperature. However, the uniform large-scale area of UHA is strongly restricted by hydrodynamic conditions along the surface and thermal exchange between the grown oxide and the environment. The abovementioned transition between HA and UHA enables the determination of stable conditions near UHA, known as pre-UHA. This offers potential application benefits by combining UHA’s high growth rate with HA’s uniform anodization.

Thus, these findings determined a rapid oxide growth rate of up to 88.3 ± 5.1 µm/h and a thicker coating of up to 234.9 ± 3.7 µm. It was also shown that the pre-anodization procedure increases the maximal pre-UHA voltage from ~130 V to ~140 V, facilitating the production of a protective oxide layer. The grown layer must protect the sample from anodic burning, enabling possible uniform anodization at a high growth rate.

## Figures and Tables

**Table 1:** Pre-anodization procedure for LSV characterization.

	40 V	60 V	80 V	100 V	120 V
P40	300 s	-	-	-	-
P60	300 s	450 s	-	-	-
P80	300 s	450 s	600 s	-	-
P100	300 s	450 s	600 s	750 s	-
P120	300 s	450 s	600 s	750 s	900 s

**Table 2:** Pore diameter at different anodization times and temperatures.

Pore Diameter (nm)					
	1 h	2 h	4 h	6 h	12 h
**0 °C**	79.9 ± 10.7	85.7 ± 9.5	88.6 ± 9.4	103.2 ± 14	98.0 ± 10.3
**5 °C**	86.4 ± 10.2	90.1 ± 15.0	96.1 ± 10.7	111.9 ± 14.3	108.7 ± 10.3
**10 °C**	102.2 ± 15.2	99.0 ± 10.9	99.0 ± 14.3	115.6 ± 16.2	109.4 ± 15.6
**15 °C**	123.6 ± 17.4	138.4 ± 14.6	139.1 ± 17.9	153.4 ± 20.6	155.9 ± 17.0

## Data Availability

The original contributions presented in the study are included in the article, further inquiries can be directed to the corresponding authors.
